# Identification and validation of protective glycoproteins in *Haemonchus contortus* H11

**DOI:** 10.3389/fimmu.2025.1521022

**Published:** 2025-02-28

**Authors:** Hui Liu, Yao Zhang, Jiarui Li, Feng Liu, Lisha Ye, Xin Liu, Chunqun Wang, Min Hu

**Affiliations:** ^1^ National Key Laboratory of Agricultural Microbiology, College of Veterinary Medicine, Huazhong Agricultural University, Wuhan, China; ^2^ College of Life Science and Technology, Huazhong University of Science and Technology, Wuhan, China

**Keywords:** H11, vaccine, glycoprotein, N-glycan, recombinant antigen, insect cells, immunoprotection

## Abstract

Barbervax is the first and only available vaccine to protect animals against *Haemonchus contortus* - one of the most pathogenic parasites of small ruminants. This vaccine contains a kind of native antigen called H11, a glycoprotein complex derived from integral gut of this parasite. Native H11 has been shown to induce high levels (72-95%) of protection, but single or two recombinant molecules of H11 are consistently unsuccessful. An increasing number of aminopeptidases related to H11 have been characterized in the past three decades, but little is known about which ones are the key contributors to protective immunity. Our recent work has revealed that the immunoprotective effect of H11 is primarily associated with its N-glycan moieties. To identify key immunoprotective glycoproteins derived from H11 antigen, we employed glycan-related protective IgG antibodies combined with LC-MS/MS analysis and identified five glycosylated H11 proteins: H11, H11-1, H11-2, H11-4, and H11-5. Subsequently, we utilized the baculovirus-insect cell expression system and successfully expressed four H11 recombinant proteins including rH11, rH11-1, rH11-2 and rH11-4, which demonstrated similar aminopeptidase activity and comparable high-mannose and di-fucosylated N-glycan structures to those found on native H11. Immunization of goats with a cocktail of four rH11s induced a 66.29% reduction (*p* > 0.05) in total worm burden and cumulative fecal egg counts. High level of anti-rH11s IgG which could inhibit *H. contortus* intestinal aminopeptidase activity and larval development. Collectively, our study identified glycoprotein antigens from H11 and assessed their protective efficacy of a recombinant cocktail expressed in insect cells. This work will provide valuable insights into further development of recombinant vaccines against parasitic nematodes.

## Introduction

Roundworms (nematodes) form one of the largest and most diverse groups of animals. Many species, including gastrointestinal nematodes (GINs), result in serious threats to the health, welfare, and productivity of grazing animals (1, 2). GINs impose severe economic losses on livestock husbandry in numerous countries all over the world (3–6). Current strategies for controlling GINs rely on anthelmintic treatments, which have become increasingly costly and complicated due to the global rise in drug resistance (1, 5, 7, 8). Therefore, there is an urgent need for new and effective integrated prevention and management strategies to control the spread of drug-resistant GIN diseases.

Vaccination is regarded as an alternative and sustainable intervention strategy for controlling GIN parasitosis in livestock. Currently, the only GIN vaccine available is Barbervax, the first effective vaccine developed to control *Haemonchus contortus*. This parasite, commonly known as the barber’s pole worm, is the most significant roundworm parasite of ruminants worldwide. This vaccine is based on intestinal membrane proteins from adult *H*. *contortus*. H11, a 110 kDa glycoprotein complex, which is the most important component in Barbervax. Administrated H11 alone could achieve > 75% reduction in worm burden and > 90% reduction in fecal egg counts (FECs) (9–13). Despite high protections induced by the native proteins, however, various recombinant forms of H11 expressed in different expression systems, such as bacteria (14), insect cells (15), and *Caenorhabditis elegans* (16, 17), have failed to provide the expected immune protection compared to native H11 (nH11).

Indeed, extensive research efforts have proven that nH11 is not a single protein but a collection of highly glycosylated proteins, containing a mixture of multiple glycoproteins from the microsomal M1 aminopeptidase family (18), which is believed to be involved in the degradation of small peptides during the digestion of host hemoglobin (19). Initially, three H11 isoforms (GenBank accession nos. AJ249941, AJ249942 and AJ311316) were cloned and sequenced based on gene analysis (14) and another isoform (GenBank accession no. X94187) was subsequently isolated using anti-H11 sera from the *H. contortus* cDNA library (18), In 2013, Roberts et al. identified a novel H11 sequence (GenBank accession no. KF381362.1) through *H. contortus* genomic data (17). With the rapid development of advanced omics techniques, an updating number of H11-related aminopeptidases have been characterized. There are 13 novel aminopeptidases (termed AP-1 to AP-13) that have been identified in the M1 aminopeptidase family through genomic and transcriptomic analyses (21), and 85 distinct proteins were identified in the N-glycoproteome of nH11 using glycoproteomic techniques (20). Moreover, as a highly glycosylated complex, our recent work demonstrated that the H11-induced immune protection was predominantly related to N-glycans (20). Nevertheless, it remains unclear which specific glycoproteins among them are the crucial contributors to immunoprotection and whether these H11 proteins exhibit redundant or synergistic immunological functions. Additionally, due to the abundant and unique N-glycan structures [e.g. core α1,3-linked fucoses, antennal fucosylated GalNAc-GlcNAc (LDNF)] present in nH11 (20), which are not commonly present in vertebrate glycans and are considered highly immunogenic (21–23). Accurately simulating these unusual glycan structures in the expression systems is another significant challenge in recombinant vaccine development.

Previously unsuccessful immune protection provided by single (14, 15, 24) or dual rH11s (17) suggests that identifying key protective glycoproteins derived from nH11 complex, preserving the integrity of glycan structures in rH11s, and fully combining them into a multivalent cocktail antigen may enhance immune protection in recombinant vaccines. Our previous study revealed that glycan-induced protective IgG antibodies confer specific and passive immunity in animals (20). Here, we identified the dominant glycoprotein antigens from nH11 by this glycan-related protective IgG antibodies. These identified glycoproteins were produced in the insect cell expression system that possesses the closest N-glycosylation pattern to nH11 among the available expression systems (25), and their aminopeptidase activities, N-glycan motifs, as well as protective effects in animals of these recombinant H11s (rH11s) were assessed. These findings have important implications for development of related parasite vaccines.

## Materials and methods

### Parasite materials

The infective third-stage larvae (iL3s) and female adult worms of *H. contortus* (Haecon-5 strain, a standard strain that has been preserved in our laboratory) were collected and maintained following previously established protocols (26). Briefly, the iL3s were isolated from feces cultured at 25°C for 7 days using the Baermann collecting procedure. To collect exsheathed L3s (xL3s) *in vitro*, the iL3s were exsheathed and sterilized by incubation in 0.15% v/v sodium hypochlorite solution at 37°C for 10 min, followed by centrifugation (1,000 *g*, 3 min) to remove sheaths. Female adult worms were collected from the abomasa of goats and distinguished according to morphological characteristics, such as red and white spiral stripes.

### Immunoprecipitation

Four kinds of serum IgG antibodies used in immunoprecipitation assay were from our previous study (20), derived from four groups of goats vaccinated with three different H11 antigens (NA-native H11, DN-heat denatured H11 and PI-periodate treated H11) and adjuvant (AJ), respectively. Briefly, 50 μg of each IgG antibodies (NA, DN, PI and AJ) were added to 50 μL of Protein A+G magnetic beads (Beyotime Biotechnology) pre-washed with cold phosphate-buffered saline (PBS; pH 7.4) and incubated at 4°C for 2 h. Following incubation, a magnetic stand was employed to separate the beads, and unbound IgG antibodies were removed through multiple washes with cold PBS. Subsequently, 100 μg of nH11, extracted using Con A-Sepharose (GE Healthcare) as described previously (9, 20), was added to each of the four groups of IgG antibodies and incubated at 4°C for 3 h to ensure complete binding of nH11 to the antibodies. Post-incubation, the beads were subjected to magnetic separation for 15 s, followed by the removal of the supernatant and thorough washing with PBS. The eluate was then analyzed by SDS-PAGE and reserved for subsequent LC-MS/MS analysis.

### LC-MS/MS analysis

Four different sets of immunoprecipitated antigen-antibody complexes were thoroughly washed with 50 mM NH_4_HCO_3_ solution and subsequently incubated in 100% acetonitrile. The complexes were then rehydrated with trypsin at a concentration of 10 ng/μL, dissolved in 50 mM NH_4_HCO_3_ solution, and kept on ice for 1 h. Peptides were initially extracted with a solution of 50% acetonitrile and 5% formic acid, followed by extraction with 100% acetonitrile. The peptides were then completely dried and resuspended in a solution of 2% acetonitrile containing 0.1% formic acid. After preparation, the peptides were analyzed using a nano-spray ionization (NSI) source, followed by tandem mass spectrometry (MS/MS) on a Q Exactive™ Plus mass spectrometer (Thermo Fisher Scientific), which was coupled online to an ultra-performance liquid chromatography (UPLC) system. An electrospray voltage of 2.0 kV was applied, with a mass-to-charge (*m/z*) scan range set between 350 and 1,800 for full-scan analysis. Intact peptides were detected in the Orbitrap at a resolution of 70,000. Selection for MS/MS analysis was conducted with a normalized collision energy (NCE) setting of 28, and fragment ions were detected in the Orbitrap at a resolution of 17,500. A data-dependent acquisition method was employed, alternating between one MS scan followed by 20 MS/MS scans, incorporating a dynamic exclusion duration of 15 s. The automatic gain control (AGC) target was set to 5E4.

The resultant MS/MS data were processed using Proteome Discoverer 1.3 software. Tandem mass spectra were queried against the *H. contortus* database (UniProt, 24,551 sequences). Trypsin/P was designated as the proteolytic enzyme, permitting up to two missed cleavages. The precursor ion mass tolerance was set at 10 ppm, while the fragment ion mass tolerance was set at 0.02 Da. Carbamidomethylation of cysteine residues was specified as a fixed modification, and methionine oxidation was defined as a variable modification. Peptide identification confidence was set to high, with a peptide ion score threshold of >20.

### Sf9 and High Five insect cells culture

Sf9 cells (Invitrogen) employed for recombinant virus production were maintained as adherent cultures at 28°C in Sf-900™ II medium (Thermo Fisher Scientific). High Five cells (Invitrogen) used for protein expression were maintained in suspension in cell shakers at 120 rpm at 28°C in Express Five™ medium (Thermo Fisher Scientific). Both cell lines were passaged every three days. Cell density was determined by Malassez hemocytometer (Marienfeld), and cell viability was assessed by Trypan blue staining (1 mg/mL, v/v).

### Cloning, protein expression and purification of H11 molecules

To express the recombinant proteins in insect cells, five H11 coding sequences (GenBank accession nos. AJ249941.1, AJ249942.2, AJ311316.1, KF381362.1, Q10737 of H11-1, H11-2, H11-4, H11-5 and H11, respectively) were synthesized based on insect cell codon bias and subcloned into a baculovirus vector, pFastBac1, using BamHI and HindIII restriction sites. The original transmembrane sequence was removed, and the product was cloned downstream of an additional signal sequence (MKTIIALSYIFCLVFAAG) in the pFastBac1 expression vector, along with an N-terminal Flag tag and 10 × His tag for identification and purification. Diagrammatic representations of final expression constructs were shown in **Supplementary Figure 1**. Protein expression and purification followed well-established protocols. In brief, Sf9 cells were seeded onto six-well plates at 8 × 10^5^ cells/mL and incubated in Sf-900™ II medium (Thermo Fisher Scientific) containing 1.5% fetal bovine serum (FBS; Invitrogen) at 28°C for 24 h before transfection. 1 μg of baculovirus plasmid was co-transfected with 8 μL of Cellfectin™ II Reagent (Thermo Fisher Scientific) into Sf9 cells according to the manufacturer’s instructions. The transfection mixture was removed 5 h post-transfection and replaced with 2 mL of Sf-900™ II medium supplemented with 10% FBS. Sf9 cells were then cultured under the same conditions for 96 h, and then the transfection supernatant was harvested and amplified twice to obtain a high titer of recombinant virus. High Five cells were infected with the recombinant virus at a multiplicity of infection (MOI) of 5 in the exponential growth phase (1×10^6^ cells/mL; 95% viability) in shake flasks at 28°C for 96 h. The culture media was collected and centrifuged at 3,000 *g* for 15 min, then purified utilizing affinity chromatography with Ni Sepharose™ excel (GE Healthcare) following eluted with 200 mM imidazole. The harvested eluent was dialyzed against PBS for 48 h and finally concentrated with sucrose. The concentrations of the final purified proteins were measured with the BCA Protein Assay Kit (Vazyme) and the purity was verified by SDS-PAGE staining with Coomassie brilliant blue.

### Aminopeptidase activity assay of recombinant H11

Aminopeptidase catalyzes the conversion of the substrate L-leucine-p-nitroanilide (L-Leu-pNA) to yield the compound p-nitroaniline (pNA). The concentration of pNA, determined by absorbance at 405 nm, serves as an indicator of total aminopeptidase activity. To evaluate whether these rH11s exhibit aminopeptidase activity and to determine their optimal pH, 10 μg of each rH11 was incubated with 10 μL of 0.2 mM L-Leu-pNA (Sigma) at 37°C for 150 min at a shaking speed of 100 rpm. Phosphate buffers with varying pH values (ranging from 4.0 to 8.0) were added to achieve a final volume of 100 μL in 96-well microtiter plates. Absorbance was recorded at 405 nm using a multi-mode plate reader (BioTek Cytation 5), and the rate of optical density change per minute per microgram of protein was subsequently calculated.

### Release, purification and permethylation of N-glycans of recombinant H11

The N-glycan preparation procedure was conducted as described previously (20). Briefly, 50 μg of each rH11 was denatured with 1 × glycoprotein denaturing buffer (0.5% SDS, 40 mM DTT) at 100°C for 10 min. Following the addition of NP-40 and GlycoBuffer 2 (50 mM sodium phosphate; pH 7.5), 2 μL of PNGase F (New England Biolabs) was added, and the reaction mixture was incubated overnight at 37°C. The glycan supernatant was isolated by centrifugation at 12,000 *g* for 15 min. To fully release all N-glycans, the remaining glycoprotein was washed, dried, denatured, and dissolved in GlycoBuffer 3 (50 mM sodium acetate; pH 6.0). Next, 2 μL of PNGase A (New England Biolabs) was added, and the reaction mixture was incubated at 37°C for 6 h. The glycan supernatant was harvested as above. Glycans released by PNGase F and PNGase A were further purified by porous graphitic carbon (PGC) cartridges, and the collected N-glycan eluent was dried at 37°C in a centrifugal evaporator. Permethylation was performed by published methods (27). Briefly, the dried N-glycan samples were dissolved in 50 μL of dimethyl sulfoxide (DMSO), followed by the addition of 100 μL of NaOH-DMSO suspension. Subsequently, the suspension was gently mixed, and 50 μL of methyl iodide was added. The mixture was fully vortexed for 20 min at room temperature and terminated by adding 500 μL of ultrapure water. The final methylated glycans were isolated by extraction with water/chloroform and the chloroform layer was dried as above.

Mass spectra were acquired utilizing the 5800 MALDI-MS (SCIEX, Concord) instrument operating in positive ionization mode. The dried glycan residues were reconstituted in 15 μL of 50% methanol, and the matrix solution was prepared by dissolving 10 mg/mL 2,5-dihydroxybenzoic acid (DHB) in 50% acetonitrile supplemented with 10 mM sodium acetate. A 1:1 mixture of permethylated glycan and DHB matrix (1 μL each) was applied to a MALDI plate and crystallized. Each sample spot received 1,000 laser shots. MS/MS was conducted with air as the CID gas at 2 kV. The resulting data were processed using Data Explorer 4.0 (SCIEX, Concord), and spectra were annotated with GlycoWorkbench 2.1. Theoretical fragmentation lists were generated for MS/MS interpretation.

### Immunization trial

**Figure 1:**
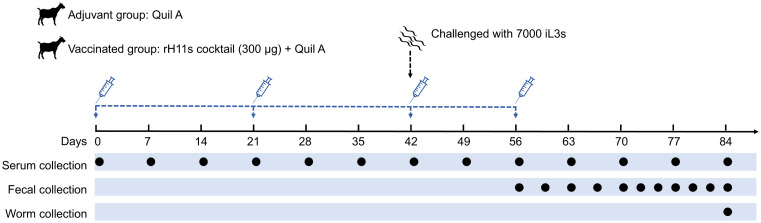
Diagram showing the procedure of animal vaccination trial. Two groups of eight goats each were immunized subcutaneously with adjuvant alone or 300 μg of rH11s cocktail four times (days 0, 21, 42 and 56). On the day of the third immunization (day 42), 7,000 infective third-stage larvae (iL3s) were challenged orally. The time points for immunization and challenge were identified with corresponding legends. Blood samples were collected weekly, and fecal samples were collected 2 to 3 times every week starting from 14 days post-challenge. On 28 days post-challenge, as the adult worms began to stably lay eggs, the frequency of fecal sample collection increased to 3 to 4 times every week. On day 84, all goats were euthanized, and the abomasa were dissected to collect adult parasites. The time points for blood collection (13 times), fecal collection (11 times) and adult worm collection (once) were indicated by black circles.

To evaluate whether the cocktail consisting of four rH11s could induce protective immunity against *H. contortus*, immunization and infection experiments were conducted in goats. All goat experiments were approved by the Animals Ethics Committee of Huazhong Agricultural University (permit code: HZAUGO-2024-0006). The workflow of the animal trials was shown in **Figure 1**. Briefly, 16 healthy Boer goats, aged 6-8 months and reared under conditions ensuring the absence of helminth infections, were randomly allocated into two groups of eight, matched by age and sex. Goats in the vaccinated group were administered 300 μg of a recombinant cocktail (comprising 75 μg each of rH11, rH11-1, rH11-2 and rH11-4) formulated with 500 μg of Quil A^®^ adjuvant (InvivoGen). The adjuvant group, serving as the control, received an equivalent dose of the Quil A^®^ adjuvant alone. Each goat was immunized subcutaneously thrice at three-week intervals (**Figure 1**). Following the third immunization on day 42, each goat was orally challenged with 7,000 iL3s and was then fasted for 8 h. An additional booster immunization was supplemented on day 56. Throughout the trial, blood and fecal samples were collected as described previously (20). Briefly, blood samples were obtained weekly from day 0 to the end of the trial. FECs were performed three to four times per week from 14 days post-iL3s challenge. On day 84, all goats were euthanized, and the number of adult male and female worms present in individual abomasum was quantified.

### Western-blot analysis

To ascertain whether specific goat anti-rH11s IgG antibodies can be elicited following vaccination and whether these antibodies can recognize nH11, the following immunoblotting experiments were conducted. Serum (200 µL per goat) on day 42 was collected from blood samples and pooled, and the IgG antibodies were isolated using Protein A+G agarose (Beyotime Biotechnology) according to the manufacturer’s instructions. Both rH11s and nH11 (50 µg of each protein) were denatured in a loading buffer with DTT and subjected to SDS-PAGE on 12.5% polyacrylamide gels. After electrophoresis, the proteins were transferred to a membrane and probed with primary IgG antibodies diluted 1:1,000. Horseradish peroxidase (HRP)-conjugated donkey anti-goat IgG (Abbkine) at a dilution of 1:5,000 was used as the secondary antibody. Detection was performed using an ECL reagent (Vazyme), and the results were visualized with a Tanon Imaging System.

### Detecting the dynamics of anti-rH11s IgG antibody

The dynamics of goat anti-rH11s IgG antibodies from each animal were assessed by indirect enzyme-linked immunosorbent assay (ELISA). In brief, serum was obtained from blood samples after centrifugation at 500  *g* for 10 min at 4°C. 96-well microtiter plates were coated with a mixture of four rH11s (400 ng per well, with 100 ng of each protein) or individual rH11 (100 ng per well) diluted in carbonate buffer (50 mM; pH 9.6) and incubated at 4°C overnight, and then blocked with PBS (pH 7.4) containing 0.05% (v/v) Tween 20 and 1% (w/v) bovine serum albumin (BSA; Sigma) at 37°C for 2 h. Individual goat sera were diluted 1:2,000 and incubated at 37°C for 1 h. Subsequently, an HRP-conjugated donkey anti-goat IgG antibody (Abbkine) was applied as the secondary antibody at a dilution of 1:5,000 and further incubation at 37°C for 40 min. The assay was developed using tetramethylbenzidine (TMB) substrate at 37°C for 15 min, and the reaction was terminated by the addition of 10% H_2_SO_4_. Absorbance was measured at 450 nm using a microplate reader (BioTek Cytation 5).

### Aminopeptidase activity and larval development inhibition assays

To determine whether anti-rH11s IgG antibodies could inhibit the aminopeptidase activity of nH11 enriched in the intestine, we isolated intestines of 30 female adults collected from non-vaccinated goats and extracted the gut proteins as previously described (28). Anti-rH11s IgG antibodies were purified from the goat serum collected on day 42 post-immunization by the protein A+G affinity chromatography (Beyotime Biotechnology). A total of 10 µg of gut proteins were pre-incubated with 5 µL of purified anti-rH11s IgG antibodies (1 mg/mL) at 37°C for 40 min. Meanwhile, 10 mM bestatin (Sigma) was employed as a positive control under identical conditions. Following the pre-incubation, 10 μL of 0.2 mM L-Leu-pNA (Sigma) was added to citrate-phosphate buffer (pH 7.0) and incubated at 37°C for 150 min. Absorbance readings were taken at 405 nm using a microplate reader (BioTek Cytation 5).

To further assess whether these anti-rH11s IgG antibodies can inhibit larval development, xL3s were cultured in 24-well plates (100 xL3s per well) in 200 μL of sterile Luria-Bertani (LB) medium, supplemented with 100 IU/mL of penicillin, 100 µg/mL of streptomycin (Sigma) and 0.25 µg/mL of amphotericin (Sigma). The larvae were treated with 50 μL of anti-rH11s IgG antibodies (1 mg/mL) and incubated at 39°C with 20% CO_2_. The developmental rate was assessed on day 4 by identifying the presence of a buccal capsule, a defining characteristic of the L4s (29). Additionally, the length and width of individual L4s were measured on the same day.

### Statistical analysis

All statistical analyses were conducted using Prism 8.0 software (GraphPad), and standard deviation (SD) or standard error of mean (SEM) was calculated. Non-parametric Mann-Whitney tests were used to perform the statistical analysis of cumulative FECs and worm burden. One-way analysis of variance (ANOVA) followed by Dunnett’s test was used to compare the enzymatic activities of different rH11s as well as the inhibition of IgG antibodies. The *p < 0.05, **p < 0.01, ***p < 0.001, ****p < 0.0001 and ns (not significant) indicated the degree of statistical significance.

## Results

### Immunoprecipitation assay differentially identified five protective H11 isoforms

**Figure 2:**
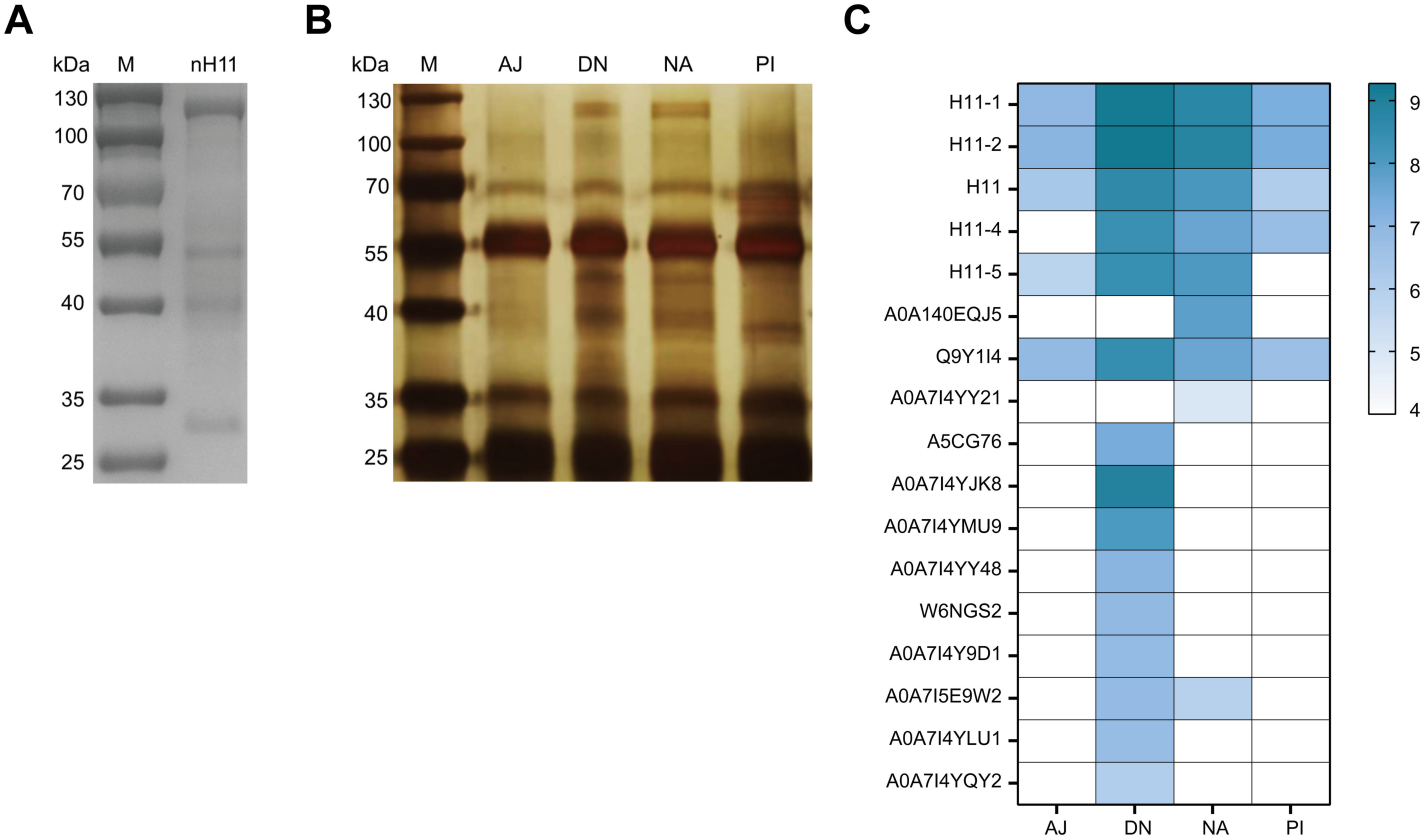
Identification of key immune protective antigens from native H11 protein complex.**(A)** SDS-PAGE analysis of nH11 purified using Con A-sepharose from *H*. *contortus* adult worms. M, protein marker. **(B)** Results of immunoprecipitation. SDS-PAGE with silver staining of nH11 proteins recognized by four groups of different IgG antibodies obtained from our previous vaccination trials in goats (20). M, protein marker. **(C)** Results of LC-MS/MS analysis. Heatmap of proteins (with molecular weights ranging from 100 to 130 kDa) abundance in different groups. AJ, IgG antibodies from adjuvant group; DN, IgG antibodies from high-temperature denatured H11 group; NA, IgG antibodies from native H11 group; PI: IgG antibodies from periodate treated H11 group.

In this study, we conducted the immunoprecipitation assay using four groups of protective IgG antibodies (NA, DN, PI, and AJ) obtained from our previous study (20) to identify key protective antigens in nH11. nH11 was extracted and purified by ConA lectin (**Figure 2A**) from *H. contortus* adult worms and then used in the immunoprecipitation assay. On the SDS-PAGE gel with silver staining following immunoprecipitation, distinct protein bands within the range of 100-130 kDa were identified by the antibodies from the NA and DN groups with high levels of immune protection (20). In contrast, this region was absent in the PI group, which exhibited a lower level of immune protection (20) as well as in the AJ control group (**Figure 2B**). To further identify the key protective H11 components differentially recognized by the above four IgG antibodies, we performed LC-MS/MS analysis against *H. contortus* databases. In *H. contortus* genomic and transcriptomic databases (30), due to the usage of different sequencing methods and *H. contortus* strains (17, 18, 31, 32), several aminopeptidases were derived from the same transcript and have different accession numbers in the UniProt database, which created the redundancies and confusion to the analysis of mass spectrometry results. To clarify this, we analyzed detailed information on 41 aminopeptidases and 42 peptidase-related proteins retrieved from *H. contortus* databases (**Supplementary Figure 2**) and reclassified them according to the latest transcriptomic data (30). The analysis results were shown in **Supplementary Table 1**. Based on these data, the differential abundances of 21 aminopeptidases (with molecular weights ranging from 100 to 130 kDa) recognized by the four groups of antibodies were identified (**Figure 2C**, **Supplementary Table 2**). The abundances of five aminopeptidases including H11-1, H11-2, H11-4, H11-5 and H11, were significantly higher in NA and DN groups than those in PI and AJ groups, suggesting these five antigens could be main contributors to the immunoprotective properties of nH11.

### Four H11 isoforms expressed in insect cells showed aminopeptidase activity

**Figure 3:**
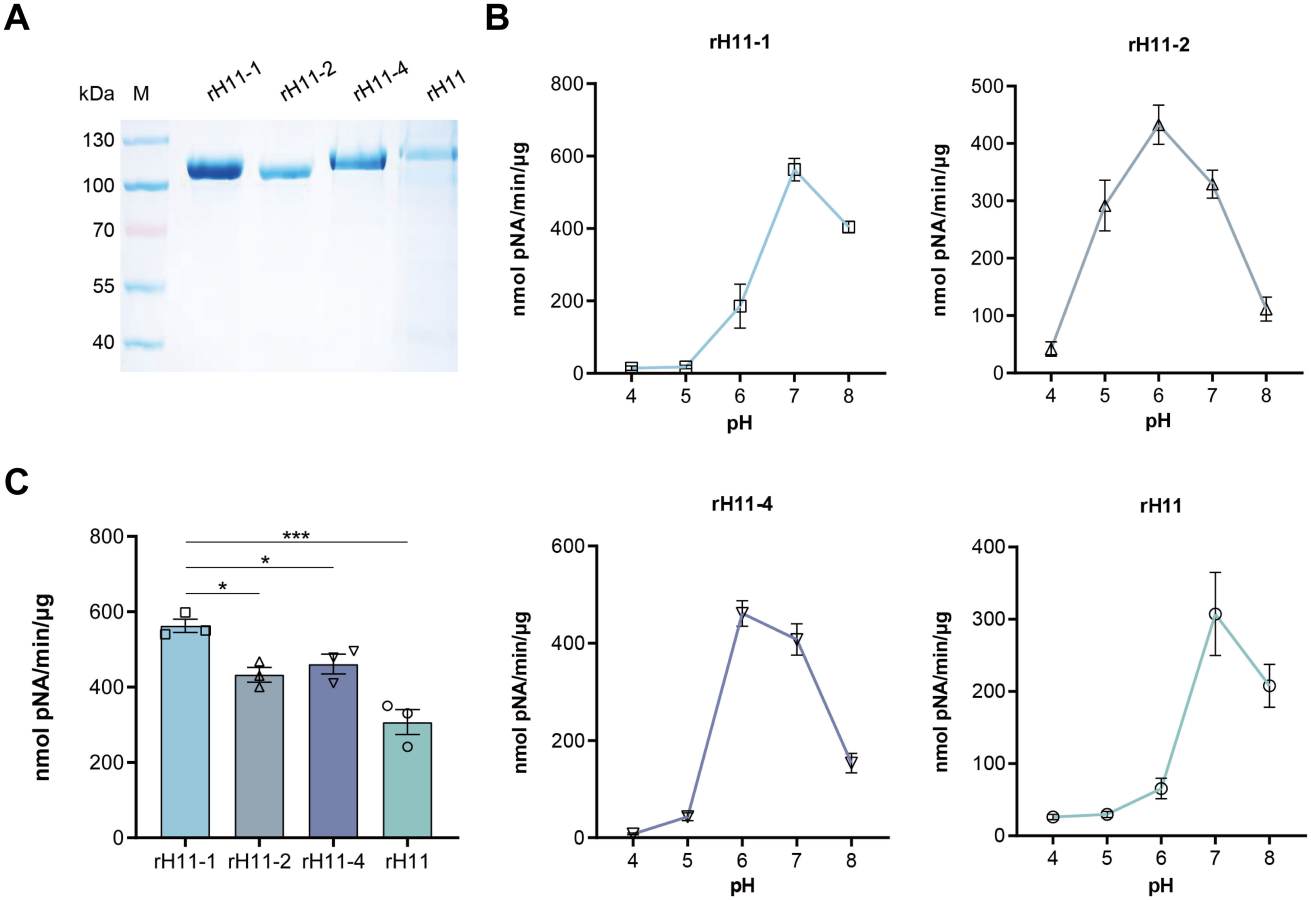
Aminopeptidase activity of four recombinant H11 proteins expressed in insect cells. **(A)** SDS-PAGE analysis of four purified rH11s expressed in insect cells. M, protein marker. **(B)** Optimal pH (ranging from 4.0 to 8.0) of aminopeptidase activity for each rH11. **(C)** Comparison of aminopeptidase activity among four rH11s. Data (absorbance at 405 nm) were pooled with the mean ± standard error of the mean (SEM) from three independent experiments, and statistically significant differences were analyzed using one-way ANOVA and indicated with *(*p* < 0.05), ***(*p* < 0.001), not significant was not marked.

We employed High Five insect cells to produce the above identified five H11 isoforms, but only four of them (H11-1, H11-2 H11-4 and H11) were successfully expressed despite multiple attempts. As expected, molecular masses of these rH11s were all around 100-130 kDa, whereas the rH11 and rH11-4 were slightly larger than those of rH11-1 and rH11-2 (**Figure 3A**), which may be associated with different protein glycosylation. To detect whether these four rH11s possess aminopeptidase activity like nH11, we assessed their aminopeptidase activities using a standard Leu-PNA substrate. All four rH11s exhibited high-level aminopeptidase activity. Both rH11-1 and rH11 exhibited optimal activity at pH 7.0 (**Figure 3B**), consistent with that of previously described native intestinal aminopeptidases (20), while rH11-2 and rH11-4 exhibited better activities at pH of 6.0 (**Figure 3B**). Notably, rH11-1 demonstrated significantly higher enzymatic activity than the other three rH11s (**Figure 3C**).

### N-glycome profiling of the recombinant H11 isoforms

**Figure 4:**
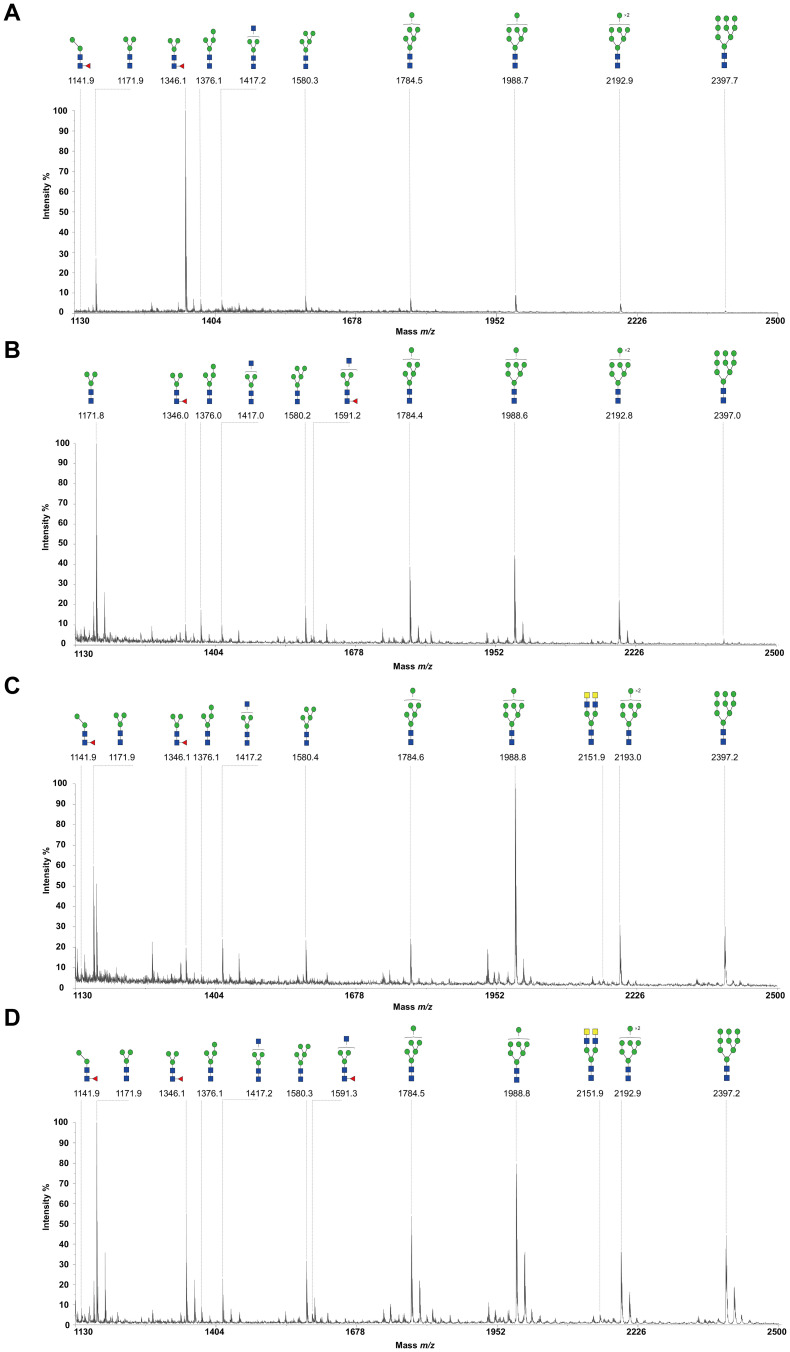
N-glycomes of four recombinant H11 proteins released by PNGase F digestion. MALDI-TOF-MS spectrum of the permethylated N-glycans of **(A)** rH11-1, **(B)** rH11-2, **(C)** rH11-4, and **(D)** rH11 released by PNGase F. Glycan species were presented primarily as [M + Na] ^+^ adducts. N-glycan signal peaks were annotated using the symbol nomenclature (green circle = mannose; blue square = GlcNAc; yellow square = GalNAc; red triangle = fucose). All N-glycan structures were deduced by the MALDI-TOF-MS/MS fragmentation and the current knowledge of N-glycomes of nH11 (20) and High Five cells (25).

**Figure 5:**
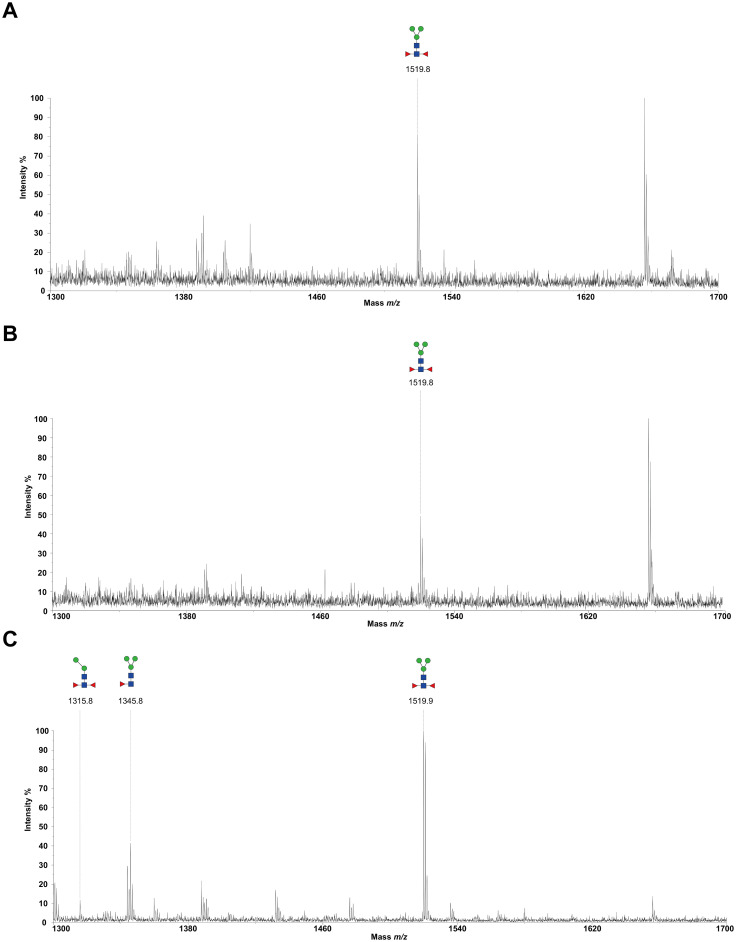
N-glycomes of three recombinant H11 proteins released by PNGase A digestion. MALDI-TOF-MS spectrum of the permethylated N-glycans of **(A)** rH11-2, **(B)** rH11-4, and **(C)** rH11, released by PPNGase A. Glycan species were presented primarily as [M + Na] ^+^ adducts. N-glycan signal peaks were annotated using the symbol nomenclature (green circle = mannose; blue square = GlcNAc; red triangle = fucose). All N-glycan structures were deduced by the MALDI-TOF-MS/MS fragmentation and the current knowledge of N-glycomes of nH11 (20) and High Five cells (25).

To decode the N-glycomes of rH11s, we conducted MALDI-TOF/MS analysis on rH11s’ N-glycans motifs. Based on the known N-glycomes data of nH11 and High Five cells (20, 25), a total of 15 glycan signal peaks, including 12 PNGase F-released glycans and three PNGase-A released glycans, were identified from four rH11s (**Figures 4**, **5**, **Table 1**). We observed that the most abundant glycans were oligomannosidic (Hex_5–9_HexNAc_2_) and paucimannosidic forms (Hex_2-3_HexNAc_2_) with and without core fucoses (**Figures 4**, **5**, **Table 1**). The majority of N-glycans were entirely released following treatment with the PNGase F, and the N-glycans released by the PNGase A served as additional components post-PNGase F treatment, as it can specifically cleave the core α1,3-linked fucose motif. rH11-2, rH11-4 and rH11 displayed characteristic N-glycan signal peaks after PNGase A digestion, predominantly featuring the Hex_3_HexNAc_2_Fuc_2_ configuration (*m/z* 1519.9), indicating the presence of di-fucosylated glycan forms (**Figure 5**, **Table 1**). In rH11, two distinct α1,3-linked fucose glycans, Hex_2_HexNAc_2_Fuc_1_ (*m/z* 1315.8) and Hex_3_HexNAc_2_Fuc_1_ (*m/z* 1345.8) were additionally identified (**Figure 5C**, **Table 1**), which were absent in the other three rH11s (**Figures 5A, B**, **Table 1**). Besides, the unusual LDN glycans (*m/z* 2151.9) existed in rH11-4 and rH11 N-glycomes, although their abundance was limited (**Figures 4C, D**, **Table 1**).

**Table 1:** N-glycan structures and abundances for four recombinant H11 proteins released by PNGase F and PNGase A.

NO.	Glycan	Composition	*m/z* [M + Na]^+^	Relative abundance (%) ^a^			
				rH11-1	rH11-2	rH11-4	rH11
1	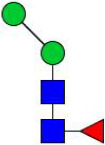	Hex_2_HexNAc_2_Fuc_1_	1141.90	3.50	1.38	2.39	1.67
2	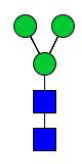	Hex_3_HexNAc_2_	1171.92	33.09	13.45	13.88	17.38
3	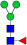	Hex_3_HexNAc_2_Fuc_1_	1346.10	4.07	54.28	5.69	9.74
4	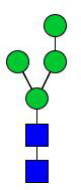	Hex_4_HexNAc_2_	1376.15	5.79	4.23	2.21	1.72
5	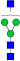	Hex_3_HexNAc_3_	1417.19	4.01	4.15	7.36	4.48
6	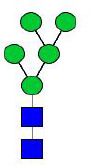	Hex_5_HexNAc_2_	1580.00	7.07	5.18	7.25	6.87
7	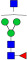	Hex_3_HexNAc_3_Fuc_1_	1591.37	1.42	2.61	–	1.14
8	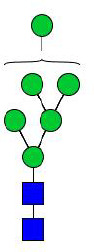	Hex_6_HexNAc_2_	1784.56	14.52	5.00	7.07	13.07
9	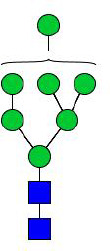	Hex_7_HexNAc_2_	1988.79	17.07	5.88	34.37	21.61
10	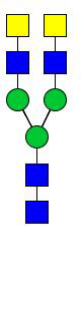	Hex_3_HexNAc_6_	2151.94	–	–	0.97	1.22
11	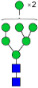	Hex_8_HexNAc_2_	2192.99	8.39	3.13	9.83	10.03
12	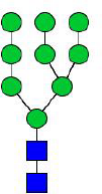	Hex_9_HexNAc_2_	2397.22	1.06	0.70	8.97	11.07
13	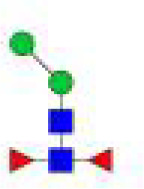	Hex_2_HexNAc_2_Fuc_2_	1315.85	–	–	–	7.17
14	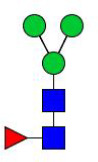	Hex_3_HexNAc_2_ Fuc_1_	1345.84	–	–	–	26.58
15	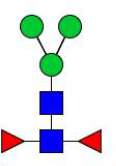	Hex_3_HexNAc_2_Fuc_2_	1519.90	–	100	100	66.25

a Relative abundance of each glycan (%) = (Peak area of each glycan)/(Total of peak of all glycans) × 100%. The relative abundances of glycans released by PNGase F and PNGase A were calculated separately. Nos. 1-12: N-glycans of recombinant H11 proteins released by PNGase F; Nos. 13-15: N-glycans of recombinant H11 proteins released by PNGase A. N-glycan structures were annotated using the symbol nomenclature (green circle = mannose; blue square = GlcNAc; yellow square = GalNAc; red triangle = fucose).

### Protective effect of recombinant H11 cocktail antigens on goats

To examine the immune protective effectiveness of this rH11s cocktail, we designed an animal vaccination trial as depicted in **Figure 1**. Between day 18 and day 21 post-challenge, both groups of goats began to excrete *H. contortus* eggs in feces. The result revealed that the mean cumulative FECs of the adjuvant control group continuously increased, peaking at 1,600 on day 40. In contrast, the mean cumulative FECs of goats vaccinated with rH11s remained below 550 over the course of trials. The reduction in mean cumulative FECs ultimately reached 66.29% compared to the adjuvant group (**Table 2**). Upon slaughter on day 42 post-challenge, the vaccinated group achieved a 66.29% reduction in total worm burden (**Table 2**), however, the reductions in both cumulative FECs and worm burden between the two groups were not statistically significant (*p > 0.05*).

**Table 2:** Fecal egg counts and worm burden of goats in the vaccination trial.

Group	Cumulative fecal egg count (FEC)				Intensity of infection			
	FEC	Mean	SD	Reduction^b^ (%)	Worm count	Mean	SD	Reduction^b^ (%)
AJ (*n* = 8)	600	9887.50	10651.28	Not applicable	5	238.63	253.80	Not applicable
	900				30			
	3150				44			
	4900				212			
	5500				187			
	9550				138			
	22350				775			
	32150				518			
Vaccinated (*n* = 7) ^a^	750	3332.85	2818.54	66.29	16	80.43	59.31	66.29
	850				36			
	950				44			
	2400				83			
	3700				145			
	5930				49			
	8750				190			

Fecal samples were taken from individual goats at 11 time points from 14 days post-challenge (cf. **Figure 1**), and the number of *H. contortus* eggs per gram of fecal samples (Fecal egg count, FEC) was counted using the McMaster counting method. Cumulative FECs of individual goats and mean cumulative FECs (with standard deviations, SD) were calculated for each group. At the end of the experiment (day 84), the total worm numbers and mean worm numbers (with standard deviations, SD) in the abomasa were counted, and the reduction in the intensity of infection was calculated for each group.

^a^ One goat died of a cause unrelated to haemonchosis on day 30. ^b^ The reduction (%) = 100 – [the mean value for vaccinated group ÷ mean value for adjuvant control (AJ) × 100%]. *n*: the number of goats. Statistical significance was determined by Mann - Whitney U-tests.

### rH11s induced high-level specific IgG antibody

**Figure 6:**
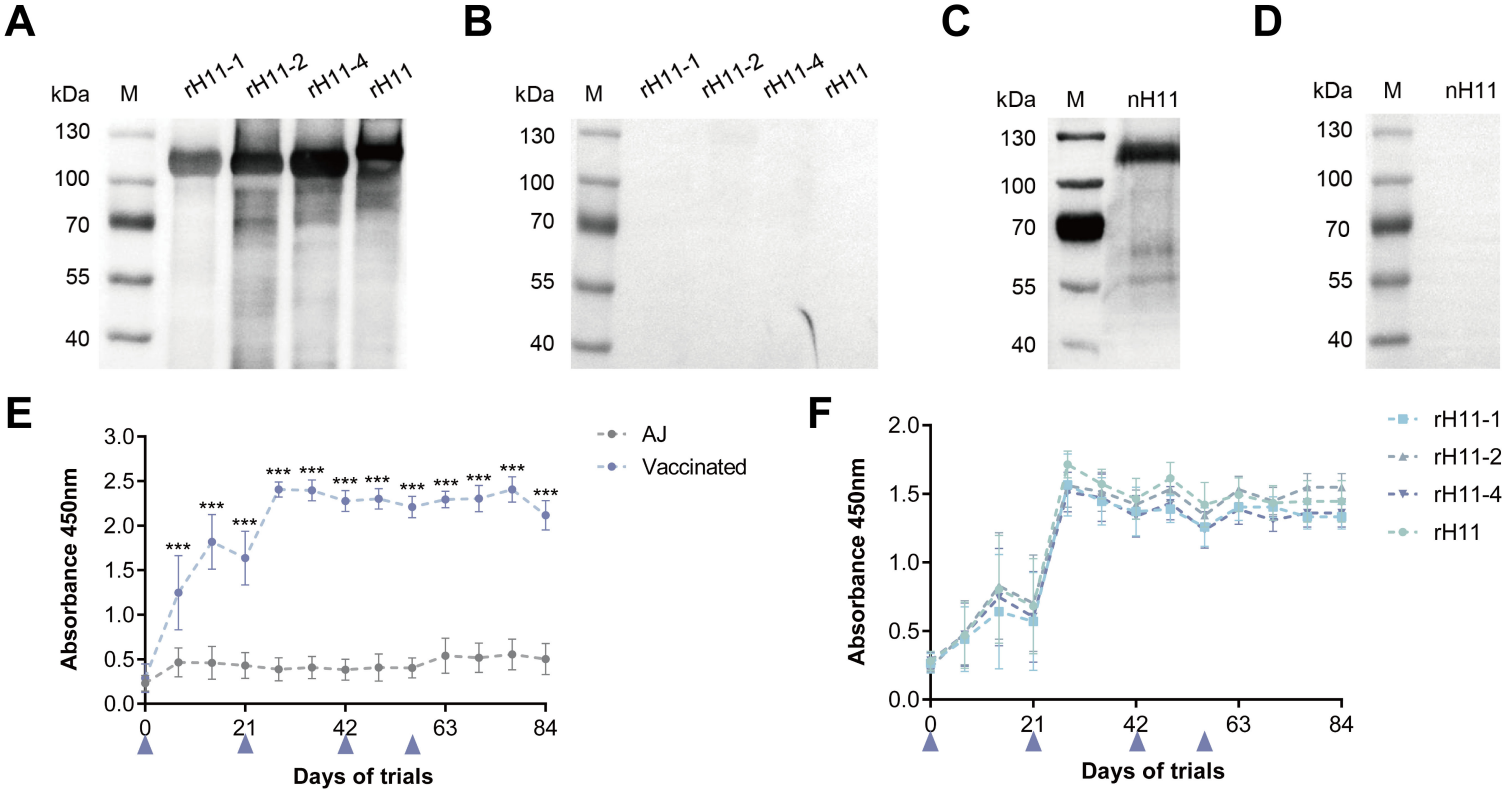
Serum IgG antibody response in goats vaccinated with recombinant H11 proteins. Western blot analysis showing serum IgG antibodies from the goats of the **(A)** vaccinated group and the **(B)** control group interacting with each of the four rH11s. Western blot analysis showing serum IgG antibodies from the goats of **(C)** vaccinated group and **(D)** control group interacting with nH11. IgG antibodies were isolated from serum samples collected from different groups on day 42 of the vaccination trial, and probed at a dilution of 1:1,000. **(E)** The dynamics of serum IgG antibodies from both vaccinated and control groups interacting with cocktail rH11s. **(F)** The dynamics of serum IgG antibodies from the vaccinated group interacting with each of the recombinant H11 proteins. Each data point (absorbance at 450 nm) represented the mean group antibody titers (mean ± SD; adjuvant control group, *n* = 8; vaccinated group, *n* = 7). The blue triangles indicated the four time points of vaccine administration. Statistically significant differences were assessed using Mann-Whitney U-tests, with significance denoted by ***(*p* < 0.001), not significant was not marked.

Previous studies have shown that the excellent immune protective properties of nH11 are primarily mediated by IgG antibodies (9, 20, 33). To determine whether immunization with rH11s induces an antibody-mediated immune response, we isolated IgG antibodies from serum samples of two groups following the initial immunization. Immunoblotting results showed that antibodies from the immunized group specifically targeted four rH11s (**Figure 6A**), in contrast to those from the adjuvant group (**Figure 6B**). Furthermore, we demonstrated that anti-rH11s IgG antibody could obviously recognize the nH11 compared with the antibody from the control group (**Figures 6C, D**). We subsequently monitored the dynamic variation of IgG antibody levels throughout the trials between these two groups using indirect ELISA. Goats administered with rH11s showed significantly higher IgG antibody titers (*p <* 0.001) against the tested rH11s compared to those in the control group following the first vaccination, peaking on day 28 and sustaining high levels during the whole infection period (**Figure 6E**). In addition, we also examined antibody levels in response to each rH11, and there were no significant differences among four rH11s (**Figure 6F**).

### Anti-rH11s IgG antibody inhibited aminopeptidase activity and larval development

**Figure 7:**
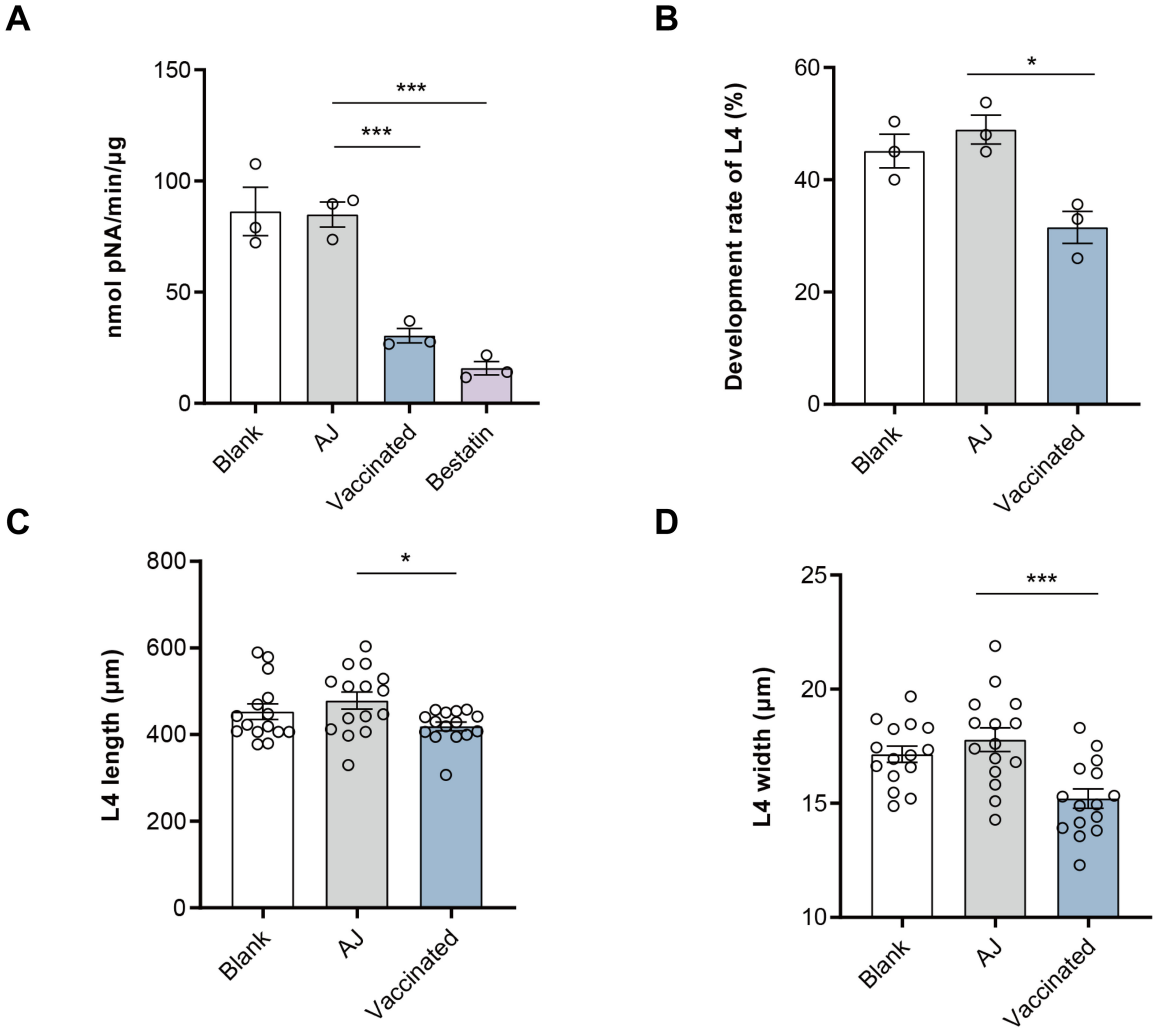
Anti-recombinant H11 IgG antibodies inhibited intestinal aminopeptidase activity and *in vitro* larval development of *Haemonchus contortus*. **(A)** Anti-rH11s antibodies inhibited intestinal aminopeptidase activity of adult worms. Inhibition assays were performed in a phosphate buffer, optimized to a pH of 7.0. Blank: IgG antibodies isolated from pre-immunization serum on day 0; AJ: IgG antibodies isolated from serum samples in the adjuvant group collected on day 42; Vaccinated: IgG antibodies isolated from serum samples in rH11s vaccinated group collected on day 42; Bestatin: a specific inhibitor of aminopeptidase. **(B)** Anti-rH11s antibodies inhibited the developmental rate of xL3s to L4s, and **(C)** the length (*n* = 15), and **(D)** the width (*n* = 15) of L4s developed *in vitro* on day 7 in the LB media containing anti-rH11 antibodies (vaccinated) or antibodies from the adjuvant group (AJ). Statistically significant differences were analyzed using one-way ANOVA and indicated with *(*p* < 0.05), ***(*p* < 0.001), not significant was not marked.

The effective protection of nH11 has been proven to be mediated by serum antibodies, whereby the parasite ingests host blood containing anti-H11 antibodies, which in turn inhibit the aminopeptidase activity and ultimately disrupt the digestion and absorption of nutrients (34, 35). In this study, we investigated whether the IgG antibodies generated against rH11s exhibit comparable inhibitory effects. The aminopeptidase activity of the native intestinal extract was significantly (*p <* 0.001) inhibited after incubation with serum IgG antibodies from the vaccinated group (30.45 nmol pNA/min/μg) compared to the adjuvant group (84.89 nmol pNA/min/μg) (**Figure 7A**). Furthermore, we showed that the inclusion of IgG antibodies from vaccinated goats at a final concentration of 0.25 mg/mL in LB medium for 7 days inhibited the development rate of xL3s to L4s compared to the control group (**Figure 7B**), as well as reduced the body length (**Figure 7C**) and width (**Figure 7D**) of L4s *in vitro*.

## Discussion

In the present study, we utilized four kinds of IgG antibodies obtained from our previous study (20), which conferred distinct immunoprotective effects to screen and identify five glycoproteins from the nH11. We subsequently expressed four recombinants by insect cells, and confirmed that these rH11s possessed similar aminopeptidase activities and N-glycan motifs to nH11. Vaccination with a cocktail of the rH11s in goats gave a 66.29% reduction in both worm burden and cumulative FECs against *H. contortus* infection although there was no significantly statistical difference. We further demonstrated that this recombinant cocktail induced high-level anti-rH11 IgG antibodies which could bind to nH11 and inhibit the aminopeptidase activity of *H. contortus* adult worm’s intestine as well as the *in vitro* larval development.

Protective efficacies of recombinant forms of one (14–16, 24) or two (17) of the above identified H11 isoforms have been tested in previous studies, but they barely induced immune protection. One of the primary reasons for the significantly decreased protective efficacy may be the exclusion of key immunogenic antigens in these recombinant vaccines, as the antigenic epitopes among H11 proteins may differ and potentially work synergistically to enhance the immune response. Cocktail vaccines against helminth infections have been proven to induce broader and more robust immune responses (36, 37). In our study, a cocktail comprising four rH11s was able to induce specific anti-rH11s IgG antibodies that were consistently maintained at high levels throughout the challenge infection and bound to both nH11 and rH11s. In contrast, a previous study reported that sheep immunized twice with the single rH11-1 expressed in insect cells displayed declining antibody responses at the eighth week post-immunization (15). Similarly, in another study, antibody levels induced by three immunizations with dual rH11s (rH11-4 + rH11-5) expressed in the free-living nematode *C. elegans* were not sustained throughout the trial, showing a significant decrease starting from the seventh week post-immunization (17). In addition to differences in the composition of recombinant vaccines, the duration of antibody responses observed in previous studies and ours may also be attributed to the number of immunizations. This suggests that further recombinant vaccine immunization strategies should emphasize the importance of booster immunizations. Regarding the immune protection induced by the nH11, it is believed that the anti-nH11 antibodies, upon ingestion with the blood meal, inhibit aminopeptidase activity and disrupt nutrient absorption (18, 20). In the present study, the anti-rH11s IgG antibodies could inhibit the aminopeptidase activities of adult worm’s intestine and the larval growth and development, which was similar to the inhibition effect of the anti-nH11 IgG antibodies (20), suggesting that this cocktail vaccine could induce a sustained and protective antibody response. Our recombinant cocktail provided a 66.29% reduction in both worm burden and cumulative FECs in goats, this result has surpassed the efficacy (<30%) of previous rH11 that utilized either single (14–16) or dual rH11s (17) although there was no statistically significant difference. We speculated that individual variations among goats affected the statistical significance. These differences may result from genetic, immune, or environmental conditions affecting responses to infection and vaccination. To address this in future studies, grouping animals based on their immune responses or genetic traits may reduce variability, while increasing the sample scale and controlling environmental variables may improve the statistical power. Despite the lack of statistical significance, the trend toward increased protection suggests that using multiple H11 immunodominant components may further improve recombinant vaccine efficacy.

Our previous studies demonstrated that abundant and unique N-glycans in the nH11 play a decisive role in the protective immunity against *H. contortus* infection (20). Notably, α1,3-linked fucose in asparagine-linked GlcNAc residues, a non-vertebrate glycosylation, is regarded as highly immunologically relevant and serves as a key epitope for IgE antibodies of *H. contortus* infection (38). In *Schistosoma mansoni*, core α1,3-fucosylated glycans widely distributed in the eggs and miracidia are also believed to play a vital role in inducing the Th2 anti-parasite immune response (39–41), and IgG against these epitopes can kill schistosomula by a complement-dependent process *in vitro* (42). Besides being widely distributed in N-glycomes of helminths (20, 43–45), the crucial core fucosylated glycans are also present in insect cells (25, 46, 47), which gives them an inherent advantage for producing rH11s compared to other eukaryotic expression systems such as yeast or mammalian cells (48). Although the H11-1 isoform had been expressed in insect cells in a previous study (15), the N-glycan structures of rH11-1 were unknown. Here, we confirmed that rH11s expressed in insect cells possessed a total of 15 N-glycans and three rH11s (rH11, rH11-2 and rH11-4) exhibited both an α1,3 and an α1,6-linked fucose residue at the proximal GlcNAc which constitute an immunogenic core di-fucosylated glycans detected at *m/z* 1519.9 like nH11 (20). Except for the fucosylated core glycans, however, several more complex N-glycan unites, such as core tri-fucosylation, galactosylated fucose structures, antennal LDNF, Lewis^x^, and galactosylated LDNF structures (20), were not detected in the N-glycome of the rH11s. Although an unusual signal peak for the LDN glycan (*m/z* 2151.9) was observed in the glycome of rH11-4 and rH11, its abundance (0.97-1.22%, cf. **Table 1**) was at a low level compared to those in nH11 (20). The variations in glycan composition or abundance may explain the decreased ability of rH11s to elicit protective immunity against *H. contortus* infection.

Glycan structural difference between rH11 and nH11 is attributed to the inadequacy or absence of specific glycosyltransferases required to elongate trimmed N-glycan processing intermediates and synthesize complex end products within the exogenous expression systems. Another factor is that insect cells possess an endogenous N-glycan trimming enzyme, which specifically removes terminal β1,2-linked GlcNAc residues to antagonize N-glycan elongation and thus cannot form glycan with complex antennal structures (49–51). Currently, glycoengineering is a promising strategy to mimic the properties of glycosylation of target glycoproteins by interfering with endogenous glycosidases or introducing heterogenous glycosyltransferases (52–55). More recent efforts to engineer insect N-glycosylation pathways have focused on the creation of transgenic insect cell lines that constitutively express mammalian glycosyltransferases (56–58), enabling precise synthesis of specific N-glycans. Although the N-glycosylation pathway of *H. contortus* has not been fully elucidated, a substantial number of glycosyltransferases have been characterized in *C. elegans*, a free-living nematode that belongs to the same evolutionary clade V as *H. contortus* and likely shares similar N-glycosylation pattern (59–61). A previous study using *C. elegans* as a novel expression system achieved N-glycans of rH11-4 closer in structure to those of nH11 (17). However, as a production system, *C. elegans* is not optimal due to its limitations in the production of foreign proteins, resulting in relatively low yields and the introduction of some additional redundant modifications (e.g. phosphorylcholine) to suppress immune responses (17). Therefore, introducing specific nematode glycosyltransferases of *C. elegans* into insect cells, such as Fut-6 (to synthesize distal core α1,3-linked fucose structure) (62), GalT-1 (to synthesize the core galactose-fucose structure) (55, 63), makes it feasible to convert trimmed N-glycans into complex structures and obtain completed N-glycosylated rH11s in the exogenous expression systems. This advancement will hold significant promise for enhancing the immune protection offered by recombinant vaccines.

In conclusion, we identified five immunoprotective glycoproteins from the nH11 and expressed four of them in the insect cell expression system. These rH11s have aminopeptidase activities and similar N-glycan profiles to those of nH11. A cocktail of rH11s resulted in a 66.29% reduction in both worm burden and cumulative FECs as well as a high level of anti-rH11s IgG that could inhibit the aminopeptidase activity of *H. contortus* adult intestine and larval development *in vitro*. Our study provides valuable insights for the future development of recombinant vaccines against *H. contortus* and other related parasites.

## Data Availability

The datasets presented in this study can be found in online repositories. The name of the repository and accession number can be found below: EMBL-EBI PRIDE Archive; PXD number PXD060969.

## Supplementary material

Supplementary Figure 1 Diagrams of construction of expression plasmids for five recombinant H11 proteins.

Supplementary Figure 2 Proportional distribution of aminopeptidase proteins retrieved from databases of *Haemonchus contortus.*

Supplementary Table 1 Information on the gene ID, gene name, transcript ID and UniprotKB accession number of 41 aminopeptidase proteins retrieved from the databases of *Haemonchus contortus*.

Supplementary Table 2 Information on molecules obtained from LC-MS/MS analysis of H11 components with molecular weights between 100-130 kDa recognized by four different IgG antibodies.

## References

[1] Maurizio A Perrucci S Tamponi C Scala A Cassini R Rinaldi L. Control of gastrointestinal helminths in small ruminants to prevent anthelmintic resistance: the Italian experience. Parasitology. (2023) 150:1105–18. doi: 10.1017/s0031182023000343 PMC10801368 37039466

[2] Hempstead MN Waghorn TS Gibson MJ Sauermann CW Ross AB Cave VM. Worms and welfare: Behavioural and physiological changes associated with gastrointestinal nematode parasitism in lambs. Vet Parasitol. (2023) 324:110056. doi: 10.1016/j.vetpar.2023.110056 37897851

[3] Roeber F Jex AR Gasser RB. Impact of gastrointestinal parasitic nematodes of sheep, and the role of advanced molecular tools for exploring epidemiology and drug resistance - an Australian perspective. Parasit Vectors. (2013) 6:153. doi: 10.1186/1756-3305-6-153 23711194 PMC3679956

[4] Chagas ACS Tupy O Santos IBD Esteves SN. Economic impact of gastrointestinal nematodes in Morada Nova sheep in Brazil. Rev Bras Parasitol Vet. (2022) 31:e008722. doi: 10.1590/s1984-29612022044 36000609 PMC12136393

[5] Rose Vineer H Morgan ER Hertzberg H Bartley DJ Bosco A Charlier J. Increasing importance of anthelmintic resistance in European livestock: creation and meta-analysis of an open database. Parasite. (2020) 27:69. doi: 10.1051/parasite/2020062 33277891 PMC7718593

[6] Strydom T Lavan RP Torres S Heaney K. The economic impact of parasitism from nematodes, trematodes and ticks on beef cattle production. Animals. (2023) 13:1599. doi: 10.3390/ani13101599 37238028 PMC10215612

[7] Charlier J Bartley DJ Sotiraki S Martinez-Valladares M Claerebout E von-Samson-Himmelstjerna G. Anthelmintic resistance in ruminants: challenges and solutions. Adv Parasitol. (2022) 115:171–227. doi: 10.1016/bs.apar.2021.12.002 35249662

[8] Voigt K Geiger M Jäger M. Five past twelve - the resistance situation in small ruminant gastrointestinal nematodes in Germany. Tierarztl Prax Ausg G Grosstiere Nutztiere. (2023) 51:153–9. doi: 10.1055/a-2097-9361 37567194

[9] Smith TS Munn EA Graham M Tavernor AS Greenwood CA. Purification and evaluation of the integral membrane protein H11 as a protective antigen against *Haemonchus Contortus* . Int J Parasitol. (1993) 23:271–80. doi: 10.1016/0020-7519(93)90150-w 8496010

[10] Newton SE Munn EA. The development of vaccines against gastrointestinal nematode parasites, particularly *Haemonchus Contortus* . Parasitol Today. (1999) 15:116–22. doi: 10.1016/s0169-4758(99)01399-x 10322325

[11] Graham M Munn EA Smith TS Knox DP Oliver JJ Newton SE eds. Recombinant DNA molecules encoding aminopeptidase enzymes and their use in the preparation of vaccines against helminth infections. United Kingdom patent WO 9323542 (1993).

[12] Munn EA Smith TS Graham M Tavernor AS Greenwood CA. The potential value of integral membrane proteins in the vaccination of lambs against *Haemonchus Contortus* . Int J Parasitol. (1993) 23:261–9. doi: 10.1016/0020-7519(93)90149-s 8496009

[13] Munn EA Smith TS Smith H James FM Smith FC Andrews SJ. Vaccination against *Haemonchus contortus* with denatured forms of the protective antigen H11. Parasite Immunol. (1997) 19:243–8. doi: 10.1046/j.1365-3024.1997.d01-205.x 9364553

[14] Newton SE Meeusen EN. Progress and new technologies for developing vaccines against gastrointestinal nematode parasites of sheep. Parasite Immunol. (2003) 25:283–96. doi: 10.1046/j.1365-3024.2003.00631.x 12969446

[15] Reszka N Rijsewijk FA Zelnik V Moskwa B Bieńkowska-Szewczyk K. *Haemonchus contortus*: characterization of the baculovirus expressed form of aminopeptidase H11. Exp Parasitol. (2007) 117:208–13. doi: 10.1016/j.exppara.2007.03.018 17482594

[16] Zhou QJ Yang Y Guo XL Duan LJ Chen XQ Yan BL. Expression of *Caenorhabditis elegans*-expressed Trans-HPS, partial aminopeptidase H11 from *Haemonchus contortus* . Exp Parasitol. (2014) 145:87–98. doi: 10.1016/j.exppara.2014.08.005 25128369

[17] Roberts B Antonopoulos A Haslam SM Dicker AJ McNeilly TN Johnston SL. Novel expression of *Haemonchus contortus* vaccine candidate aminopeptidase H11 using the free-living nematode *Caenorhabditis elegans* . Vet Res. (2013) 44:111. doi: 10.1186/1297-9716-44-111 24289031 PMC4176091

[18] Smith TS Graham M Munn EA Newton SE Knox DP Coadwell WJ. Cloning and characterization of a microsomal aminopeptidase from the intestine of the nematode *Haemonchus contortus* . Biochim Biophys Acta. (1997) 1338:295–306. doi: 10.1016/s0167-4838(96)00204-x 9128148

[19] Williamson AL Brindley PJ Knox DP Hotez PJ Loukas A. Digestive proteases of blood-feeding nematodes. Trends Parasitol. (2003) 19:417–23. doi: 10.1016/s1471-4922(03)00189-2 12957519

[20] Wang C Liu L Wang T Liu X Peng W Srivastav RK. H11-induced immunoprotection is predominantly linked to N-glycan moieties during *Haemonchus contortus* infection. Front Immunol. (2022) 13:1034820. doi: 10.3389/fimmu.2022.1034820 36405717 PMC9667387

[21] Prasanphanich NS Mickum ML Heimburg-Molinaro J Cummings RD. Glycoconjugates in host-helminth interactions. Front Immunol. (2013) 4:240. doi: 10.3389/fimmu.2013.00240 24009607 PMC3755266

[22] van Die I Cummings RD. Glycan gimmickry by parasitic helminths: a strategy for modulating the host immune response? Glycobiology. (2010) 20:2–12. doi: 10.1093/glycob/cwp140 19748975

[23] Hokke CH van Diepen A. Helminth glycomics - glycan repertoires and host-parasite interactions. Mol Biochem Parasitol. (2017) 215:47–57. doi: 10.1016/j.molbiopara.2016.12.001 27939587

[24] Yan R Li X. Expression of recombinant H11 of *Haemonchus contortus* in *Pichia pastoris* . J Nanjing Agricult Univ. (2005) 28:85–9. doi: 10.7685/j.issn.1000-2030.2005.02.018

[25] Stanton R Hykollari A Eckmair B Malzl D Dragosits M Palmberger D. The underestimated N-glycomes of *lepidopteran* species. Biochim Biophys Acta Gen Subj. (2017) 1861:699–714. doi: 10.1016/j.bbagen.2017.01.009 28077298 PMC5330436

[26] Li F Lok JB Gasser RB Korhonen PK Sandeman MR Shi D. *Hc-daf-2* encodes an insulin-like receptor kinase in the barber’s pole worm, *Haemonchus contortus*, and restores partial dauer regulation. Int J Parasitol. (2014) 44:485–96. doi: 10.1016/j.ijpara.2014.03.005 PMC4516220 24727120

[27] Ciucanu I Costello CE. Elimination of oxidative degradation during the per-O-methylation of carbohydrates. J Am Chem Soc. (2003) 125:16213–9. doi: 10.1021/ja035660t 14692762

[28] Munn EA Greenwood CA. Endotube-brush border complexes dissected from the intestines of *Haemonchus contortus* and Ancylostoma caninum. Parasitology. (1983) 87:129–37. doi: 10.1017/s0031182000052471 6684761

[29] Sommerville RI. The development of *Haemonchus contortus* to the fourth stage *in vitro* . Int J Parasitol. (1966) 52:127–36. doi: 10.2307/3276403 5910446

[30] Doyle SR Tracey A Laing R Holroyd N Bartley D Bazant W. Genomic and transcriptomic variation defines the chromosome-scale assembly of *Haemonchus contortus*, a model gastrointestinal worm. Commun Biol. (2020) 3:656. doi: 10.1038/s42003-020-01377-3 33168940 PMC7652881

[31] Mohandas N Young ND Jabbar A Korhonen PK Koehler AV Hall RS. The complement of family M1 aminopeptidases of *Haemonchus contortus* - Biotechnological implications. Biotechnol Adv. (2016) 34:65–76. doi: 10.1016/j.bioteChadv.2015.10.003 26597954

[32] Zhou QJ Zhang HL Jiang XL Du AF. The gene structure and promoter region of the vaccine target aminopeptidase H11 from the blood-sucking nematode parasite of ruminants, *Haemonchus contortus* . Funct Integr Genomics. (2010) 10:589–601. doi: 10.1007/s10142-010-0172-5 20437190

[33] Ye L Zhang Y Wu S Wang Z Liu F Wang C. Immunoprotection efficacy of Con A-purified proteins against *Haemonchus contortus* in goats. Vaccines. (2022) 10:1891. doi: 10.3390/vaccines10111891 36366399 PMC9696691

[34] Knox D. Proteases in blood-feeding nematodes and their potential as vaccine candidates. Adv Exp Med Biol. (2011) 712:155–76. doi: 10.1007/978-1-4419-8414-2_10 21660664

[35] Knox DP Redmond DL Newlands GF Skuce PJ Pettit D Smith WD. The nature and prospects for gut membrane proteins as vaccine candidates for *Haemonchus contortus* and other ruminant trichostrongyloids. Int J Parasitol. (2003) 33:1129–37. doi: 10.1016/s0020-7519(03)00167-x 13678629

[36] Nisbet AJ McNeilly TN Wildblood LA Morrison AA Bartley DJ Bartley Y. Successful immunization against a parasitic nematode by vaccination with recombinant proteins. Vaccine. (2013) 31:4017–23. doi: 10.1016/j.vaccine.2013.05.026 23707168

[37] Mendez S Zhan B Goud G Ghosh K Dobardzic A Wu W. Effect of combining the larval antigens *Ancylostoma* secreted protein 2 (ASP-2) and metalloprotease 1 (MTP-1) in protecting hamsters against hookworm infection and disease caused by *Ancylostoma ceylanicum* . Vaccine. (2005) 23:3123–30. doi: 10.1016/j.vaccine.2004.12.022 15837211

[38] van Die I Gomord V Kooyman FN van den Berg TK Cummings RD Vervelde L. Core alpha1–>3-fucose is a common modification of N-glycans in parasitic helminths and constitutes an important epitope for IgE from *Haemonchus contortus* infected sheep. FEBS Lett. (1999) 463:189–93. doi: 10.1016/s0014-5793(99)01508-2 10601665

[39] Jang-Lee J Curwen RS Ashton PD Tissot B Mathieson W Panico M. Glycomics analysis of *Schistosoma mansoni* egg and cercarial secretions. Mol Cell Proteomics. (2007) 6:1485–99. doi: 10.1074/mcp.M700004-MCP200 17550893

[40] Faveeuw C Mallevaey T Paschinger K Wilson IB Fontaine J Mollicone R. *Schistosome* N-glycans containing core alpha 3-fucose and core beta 2-xylose epitopes are strong inducers of Th2 responses in mice. Eur J Immunol. (2003) 33:1271–81. doi: 10.1002/eji.200323717 12731052

[41] van Diepen A van der Velden NS Smit CH Meevissen MH Hokke CH. Parasite glycans and antibody-mediated immune responses in *Schistosoma* infection. Parasitology. (2012) 139:1219–30. doi: 10.1017/s0031182012000273 22423613

[42] Prasanphanich NS Leon K Secor WE Shoemaker CB Heimburg-Molinaro J Cummings RD. Anti-schistosomal immunity to core xylose/fucose in N-glycans. Front Mol Biosci. (2023) 10:1142620. doi: 10.3389/fmolb.2023.1142620 37081851 PMC10110957

[43] Haslam SM Coles GC Munn EA Smith TS Smith HF Morris HR. *Haemonchus contortus* glycoproteins contain N-linked oligosaccharides with novel highly fucosylated core structures. J Biol Chem. (1996) 271:30561–70. doi: 10.1074/jbc.271.48.30561 8940027

[44] Wang C Gao W Yan S Zhu XQ Suo X Liu X. N-glycome and N-glycoproteome of a hematophagous parasitic nematode *Haemonchus* . Comput Struct Biotechnol J. (2021) 19:2486–96. doi: 10.1016/j.csbj.2021.04.038 PMC8113779 34025939

[45] Paschinger K Wilson IB. Two types of galactosylated fucose motifs are present on N-glycans of *Haemonchus contortus* . Glycobiology. (2015) 25:585–90. doi: 10.1093/glycob/cwv015 PMC4414472 25740940

[46] Tomiya N Narang S Lee YC Betenbaugh MJ. Comparing N-glycan processing in mammalian cell lines to native and engineered *lepidopteran* insect cell lines. Glycoconj J. (2004) 21:343–60. doi: 10.1023/b:Glyc.0000046275.28315.87 15514482

[47] Walski T De Schutter K Van Damme EJM Smagghe G. Diversity and functions of protein glycosylation in insects. Insect Biochem Mol Biol. (2017) 83:21–34. doi: 10.1016/j.ibmb.2017.02.005 28232040

[48] Toustou C Walet-Balieu ML Kiefer-Meyer MC Houdou M Lerouge P Foulquier F. Towards understanding the extensive diversity of protein N-glycan structures in eukaryotes. Biol Rev Camb Philos Soc. (2022) 97:732–48. doi: 10.1111/brv.12820 PMC9300197 34873817

[49] Geisler C Jarvis DL. Identification of genes encoding N-glycan processing beta-N-acetylglucosaminidases in *Trichoplusia ni* and *Bombyx mori*: Implications for glycoengineering of baculovirus expression systems. Biotechnol Prog. (2010) 26:34–44. doi: 10.1002/btpr.298 19882694 PMC3624021

[50] Geisler C Aumiller JJ Jarvis DL. A fused lobes gene encodes the processing beta-N-acetylglucosaminidase in Sf9 cells. J Biol Chem. (2008) 283:11330–9. doi: 10.1074/jbc.M710279200 PMC2431071 18303021

[51] Altmann F Schwihla H Staudacher E Glössl J März L. Insect cells contain an unusual, membrane-bound beta-N-acetylglucosaminidase probably involved in the processing of protein N-glycans. J Biol Chem. (1995) 270:17344–9. doi: 10.1074/jbc.270.29.17344 7615537

[52] Dicker M Strasser R. Using glyco-engineering to produce therapeutic proteins. Expert Opin Biol Ther. (2015) 15:1501–16. doi: 10.1517/14712598.2015.1069271 PMC7100909 26175280

[53] Geisler C Mabashi-Asazuma H Jarvis DL. An overview and history of glyco-engineering in insect expression systems. Methods Mol Biol. (2015) 1321:131–52. doi: 10.1007/978-1-4939-2760-9_10 26082220

[54] van der Kaaij A van Noort K Nibbering P Wilbers RHP Schots A. Glyco-engineering plants to produce helminth glycoproteins as prospective biopharmaceuticals: recent advances, challenges and future prospects. Front Plant Sci. (2022) 13:882835. doi: 10.3389/fpls.2022.882835 35574113 PMC9100689

[55] Mabashi-Asazuma H Jarvis DL. CRISPR-Cas9 vectors for genome editing and host engineering in the baculovirus-insect cell system. Proc Natl Acad Sci U.S.A. (2017) 114:9068–73. doi: 10.1073/pnas.1705836114 PMC5576809 28784806

[56] Okada T Ihara H Ito R Nakano M Matsumoto K Yamaguchi Y. N-glycosylation engineering of lepidopteran insect cells by the introduction of the beta1,4-N-acetylglucosaminyltransferase III gene. Glycobiology. (2010) 20:1147–59. doi: 10.1093/glycob/cwq080 20554946

[57] Hollister J Grabenhorst E Nimtz M Conradt H Jarvis DL. Engineering the protein N-glycosylation pathway in insect cells for production of biantennary, complex N-glycans. Biochemistry. (2002) 41:15093–104. doi: 10.1021/bi026455d PMC3612895 12475259

[58] Hollister JR Jarvis DL. Engineering lepidopteran insect cells for sialoglycoprotein production by genetic transformation with mammalian beta 1,4-galactosyltransferase and alpha 2,6-sialyltransferase genes. Glycobiology. (2001) 11:1–9. doi: 10.1093/glycob/11.1.1 11181556

[59] Haslam SM Dell A. Hallmarks of *Caenorhabditis elegans* N-glycosylation: complexity and controversy. Biochimie. (2003) 85:25–32. doi: 10.1016/s0300-9084(03)00041-5 12765772

[60] Hanneman AJ Rosa JC Ashline D Reinhold VN. Isomer and glycomer complexities of core GlcNAcs in *Caenorhabditis elegans* . Glycobiology. (2006) 16:874–90. doi: 10.1093/glycob/cwl011 16769777

[61] Paschinger K Gutternigg M Rendić D Wilson IB. The N-glycosylation pattern of *Caenorhabditis elegans* . Carbohydr Res. (2008) 343:2041–9. doi: 10.1016/j.carres.2007.12.018 18226806

[62] Nguyen K van Die I Grundahl KM Kawar ZS Cummings RD. Molecular cloning and characterization of the *Caenorhabditis elegans* alpha1,3-fucosyltransferase family. Glycobiology. (2007) 17:586–99. doi: 10.1093/glycob/cwm023 17369288

[63] Titz A Butschi A Henrissat B Fan YY Hennet T Razzazi-Fazeli E. Molecular basis for galactosylation of core fucose residues in invertebrates: identification of *Caenorhabditis elegans* N-glycan core alpha1,6-fucoside beta1,4-galactosyltransferase GALT-1 as a member of a novel glycosyltransferase family. J Biol Chem. (2009) 284:36223–33. doi: 10.1074/jbc.M109.058354 PMC2794738 19858195

