# Supplementary figures and images for: Identification and validation of protective glycoproteins in Haemonchus contortus H11
Source: Front Immunol. 2025 Feb 28;16:1521022. doi: 10.3389/fimmu.2025.1521022 (PMC11906660; doi:10.3389/fimmu.2025.1521022)

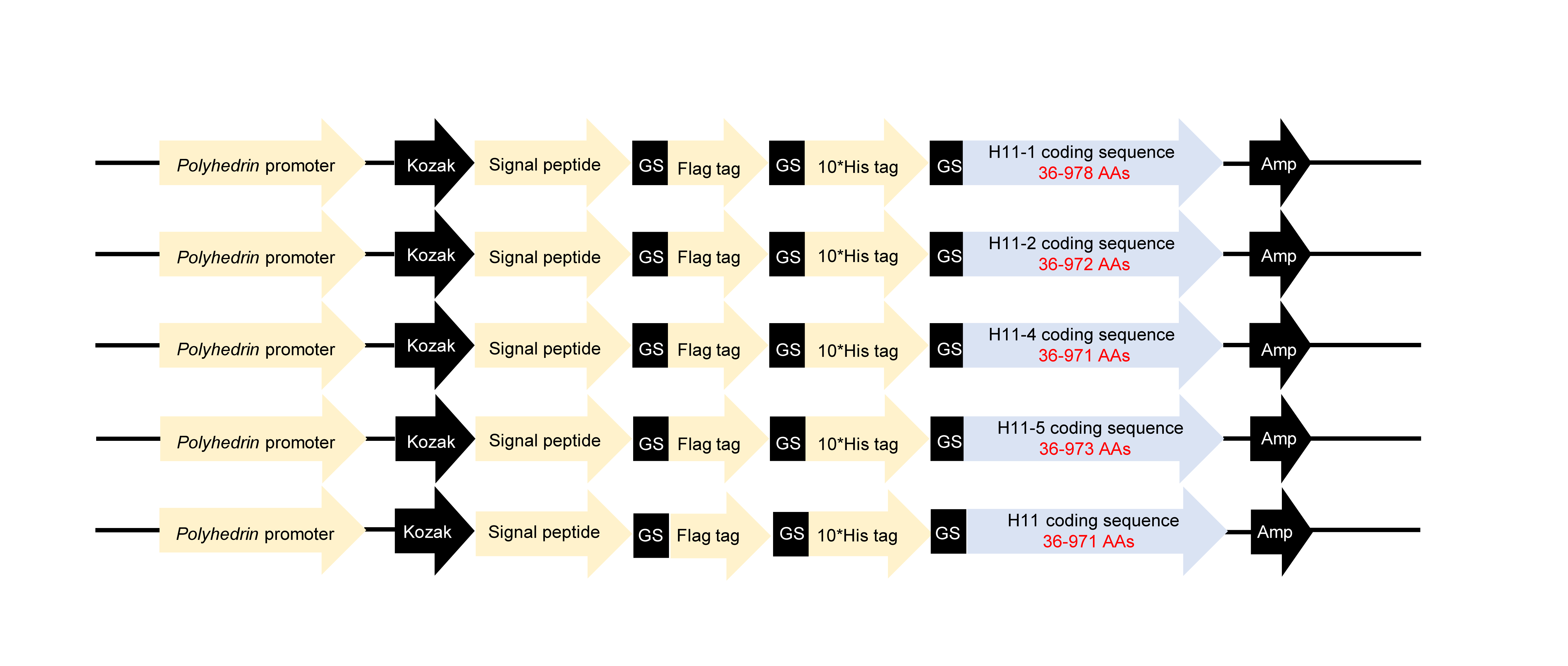

Supplement: Supplementary Figure 1 — Diagrams of construction of expression plasmids for five recombinant H11 proteins. [file Image1.tif]

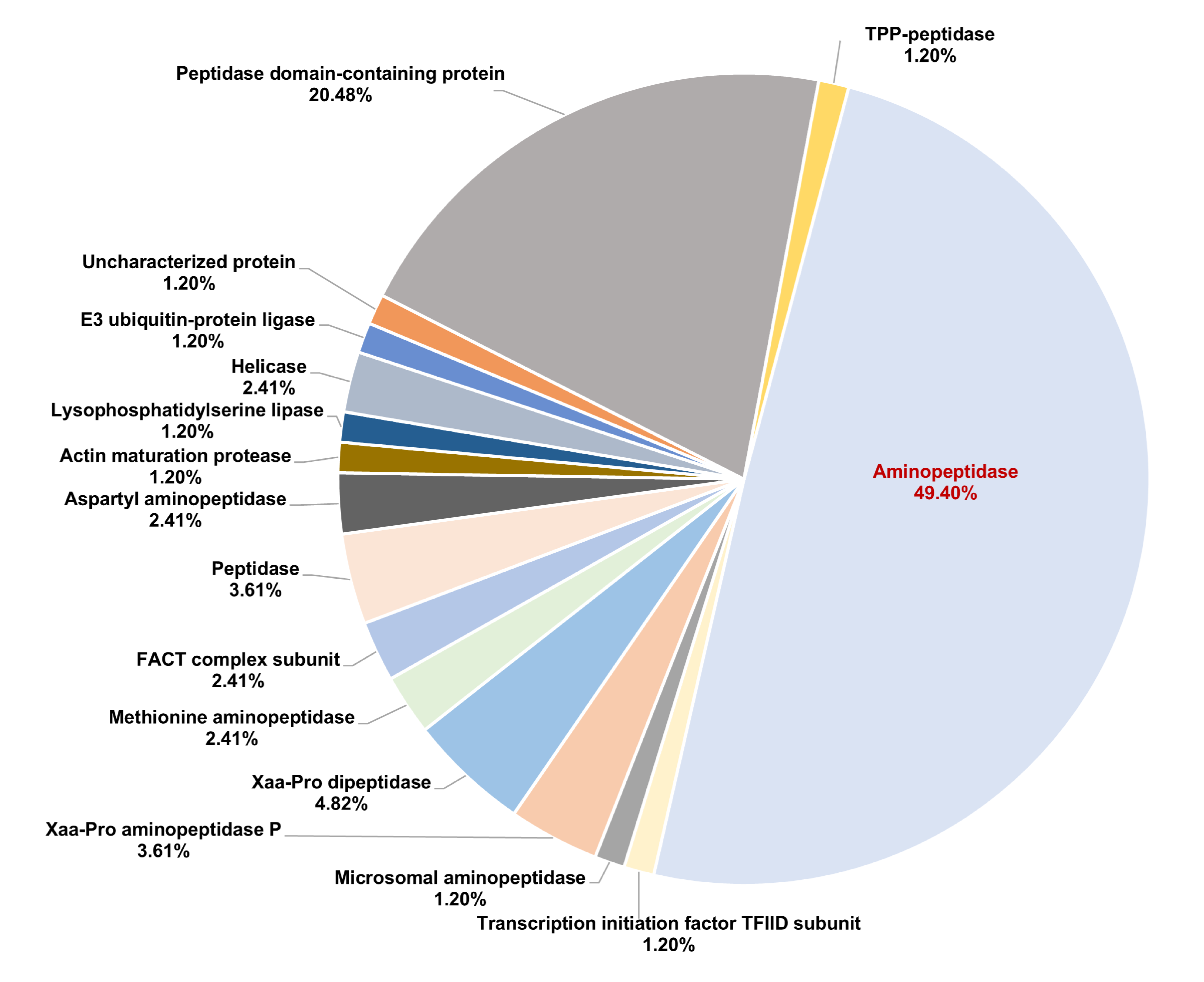

Supplement: Supplementary Figure 2 — Proportional distribution of aminopeptidase proteins retrieved from databases of Haemonchus contortus. [file Image2.tif]
