# Supplementary material for: Identification and validation of protective glycoproteins in Haemonchus contortus H11
Source: Front Immunol. 2025 Feb 28;16:1521022. doi: 10.3389/fimmu.2025.1521022 (PMC11906660; doi:10.3389/fimmu.2025.1521022)
Supplement: Supplementary Table 1 — Information on the gene ID, gene name, transcript ID and UniprotKB accession number of 41 aminopeptidase proteins retrieved from the databases of Haemonchus contortus. [file Table1.docx]

| **Gene ID** | **Gene Name** | **Transcript ID** | **UniprotKB Accession** |
| --- | --- | --- | --- |
| HCON_00156280 | h11 | HCON_00156280-00001 | A0A7I4YXA3, A0A126UAR8, A0A126UAS8, C5HLA5, Q5PY96, Q86G73, Q10737 |
| HCON_00156230 | h11-1 | HCON_00156230-00001 | A0A7I4YXT9, A0A140EQJ7, Q9U5P6, A0A7T7FPG8 |
| HCON_00156285 | h11-2 | HCON_00156285-00001 | A0A7I4YWE5, Q9U5P5, A0A7T7JNT2 |
| HCON_00156270 | h11-4 | HCON_00156270-00001 | A0A7I5ED30, A0A7T7JNU2, Q967C6 |
| HCON_00156240 | h11-5 | HCON_00156260-00001 | V5K5H8, A0A7I4YX32, A0A126UB22 |
| HCON_00095150 | ap-7 | HCON_00095150-00001 | A0A7I4YHY9, A0A140EQJ6 |
| HCON_00021510 | ap-9 | HCON_00021510-00001 | A0A140EQJ8, U6NX93 |
| HCON_00134090 | ap-11 | HCON_00134090-00001 | A0A7I4YT66, A0A140EQK0 |
| HCON_00101085 | ap-13 | HCON_00101085-00001 | A0A7I4YJF4, A0A140EQK2 |
| AML39757 | ap-3 |  | A0A126UAR9 |
| AML39758 | ap-4 |  | A0A140EQJ4 |
| AML39760 | ap-6 |  | A0A140EQJ5 |
| AML39766 | ap-12 |  | A0A140EQK1 |
| HCON_00156260 |  | HCON_00156260-00001 | A0A7I4YY48 |
| HCON_00158800 |  | HCON_00158800-00001 | A0A7I4YYR0 |
| HCON_00084300 |  | HCON_00084300-00001 | A0A7I4YD40 |
| HCON_00084350 |  | HCON_00084350-00001 | A0A7I4YE06 |
| HCON_00156250 |  | HCON_00156250-00001 | A0A7I4YY21 |
| HCON_00016690 |  | HCON_00016690-00001 | A0A7I4XXM8 |
|  |  | HCON_00016690-00002 | A0A7I4XVP0 |
| HCON_00084180 |  | HCON_00084180-00001 | A0A7I4YDZ5 |
| HCON_00142190 |  | HCON_00142190-00001 | A0A7I4YTF7 |

**SUPPLEMENTARY TABLE 1** Information on the gene ID, gene name, transcript ID and UniprotKB accession number of 41 aminopeptidase proteins retrieved from the databases of *Haemonchus contortus*
